# A fresh look at tobacco harm reduction: the case for the electronic cigarette

**DOI:** 10.1186/1477-7517-10-19

**Published:** 2013-10-04

**Authors:** Riccardo Polosa, Brad Rodu, Pasquale Caponnetto, Marilena Maglia, Cirino Raciti

**Affiliations:** 1 Presidio G. Rodolico – Unità Operativa di Medicina Interna e Medicina d’Urgenza, Centro per la Prevenzione e Cura del Tabagismo (CPCT), Azienda Ospedaliero-Universitaria “Policlinico-Vittorio Emanuele”, Università di Catania, Catania, Italy; 2 Institute of Internal Medicine, G. Rodolico Hospital, Azienda Ospedaliero-Universitaria “Policlinico-Vittorio Emanuele”, Università di Catania, Catania, Italy; 3 Department of Medicine, School of Medicine, University of Louisville, Louisville, KY, USA

**Keywords:** Tobacco, Harm reduction, Snus, Electronic cigarettes

## Abstract

Smokers of any age can reap substantial health benefits by quitting. In fact, no other single public health effort is likely to achieve a benefit comparable to large-scale smoking cessation. Surveys document that most smokers would like to quit, and many have made repeated efforts to do so. However, conventional smoking cessation approaches require nicotine addicted smokers to abstain from tobacco and nicotine entirely. Many smokers are unable – or at least unwilling – to achieve this goal, and so they continue smoking in the face of impending adverse health consequences. In effect, the status quo in smoking cessation presents smokers with just two unpleasant alternatives: quit or suffer the harmful effects of continuing smoking. But, there is a third choice for smokers: tobacco harm reduction. It involves the use of alternative sources of nicotine, including modern smokeless tobacco products like snus and the electronic cigarette (E-cig), or even pharmaceutical nicotine products, as a replacement for smoking. E-cigs might be the most promising product for tobacco harm reduction to date, because, besides delivering nicotine vapour without the combustion products that are responsible for nearly all of smoking’s damaging effect, they also replace some of the rituals associated with smoking behaviour. Thus it is likely that smokers who switch to E-cigs will achieve large health gains. The focus of this article is on the health effects of using an E-cig, with consideration given to the acceptability, safety and effectiveness of this product as a long-term substitute for smoking.

## Introduction

Tobacco smoking is a global pandemic, affecting an estimated 1.2 billion people, that poses substantial health burdens and costs. With nearly six million deaths annually, smoking is the single most important cause of avoidable premature mortality in the world [1], mainly from lung cancer, coronary heart disease, chronic obstructive pulmonary disease and stroke [2,3]. As also underscored by the World Health Organization (WHO) Framework Convention on Tobacco Control (FCTC), the key to reducing the health burden of tobacco in the medium term is to encourage cessation among smokers [4].

Unfortunately, smoking is a very difficult addiction to break, even for those with a strong desire to quit. It has been shown that approximately 80% of smokers who attempt to quit on their own relapse within the first month of abstinence, and only about 5% achieve long term abstinence [5]. Moreover, currently available smoking cessation medications such as nicotine replacement therapy, the antidepressant bupropion and the partial agonist of the α4β2 nicotinic acetylcholine receptor, varenicline, at best double or triple this quit rate under the ideal circumstances of an experimental setting but have had low uptake and inferior efficacy in the community [6-8]. Furthermore, varenicline and bupropion have come under increasing scrutiny due to reports of serious adverse events that include behaviour change, depression, self-injurious thoughts, and suicidal behaviour [9]. The Tobacco Advisory Group of the Royal College of Physicians acknowledges that the development of addiction includes modifications in behaviour together with changes in brain structure and function that impair the ability to achieve and sustain abstinence. They note that some of these changes may not be entirely reversible [10]. Lastly, even tobacco control policies - particularly when not integrated and well supported by adequate funding - are not very effective [11].

Consequently, many smokers will keep smoking because, when given only the options of smoking or completely giving up nicotine, many will not give it up. Bearing in mind that nicotine *per se* does not cause much risk when separated from inhaling smoke, it is important to consider that a third option is also available to smokers; the reduction of smoking-related diseases by taking nicotine in a low-risk form. Tobacco harm reduction (THR), the substitution of low-risk nicotine products for cigarette smoking, is likely to offer huge public health benefits by fundamentally changing the forecast of a billion cigarette-caused deaths this century [12].

### Value of harm reduction as a tobacco control strategy

The history of THR may be traced back to 1974, with the publication of a special article in the Lancet by British tobacco addiction research expert Michael A.H. Russell [13]. There are many and varied approaches to THR. Broadly, these can be categorised into two groups: (I) non-tobacco interventions aimed at decreasing tobacco consumption, and (II) alternative tobacco products. THR empowers smokers to gain control over the consequences of their nicotine addiction and at its simplest it is non intrusive and solely educational, therefore having a strong ethical rationale [14]. The strategy is cost-effective and accessible today to almost all smokers. Harm reduction is particularly compelling for nicotine because so many people have such a strong propensity for using it.

Most scientists and commentators agree that complete tobacco cessation is the best outcome for smokers, and any efforts to make available safer products need to be part of a comprehensive tobacco control strategy aimed at minimising tobacco use through cessation and prevention [15]. Opponents of THR often claim that providing safer alternatives sidetracks smokers from quitting completely. However, refusing to provide truthful information about and access to safer alternative sources of nicotine dissuades smokers from quitting the most harmful method of obtaining nicotine - inhaling smoke.

Quit rates may be improved by advancing physicians’ understanding of predictors of success in smoking cessation [16], and some have purported that it may be better to focus efforts on developing and improving pharmacologic therapies than to promote safer alternatives such as smokeless tobacco [17,18]. Currently, however, there is a growing trend in physicians’ indifference or scepticism towards the efficacy of smoking cessation programs [19]. Moreover, the use of pharmaceutical cessation aids [20] and behavioural support [21] have led to limited success in cessation, and it has been argued that the majority of current smokers will continue to smoke without acceptable safer alternatives [22]. Therefore, the case for an effective THR strategy is legitimate.

### Avoiding confusion about true health consequences of nicotine use

When considering harm reduction as a tobacco control strategy it is important to separate the risk associated with inhaling smoke from that of taking nicotine. As Russell noted 30 years ago, "There is little doubt that if it were not for the nicotine…people would be little more inclined to smoke than they are to blow bubbles or light sparklers" [13], "The rapid absorption of nicotine from snuff confirms its potential as an acceptable and relatively harmless substitute for smoking"…. "Switching from cigarettes to snuff would substantially reduce the risk of lung cancer, bronchitis, emphysema, and possibly coronary heart disease as well, at the cost of a slight increase in the risk of cancer of the nasopharynx (or oral cavity in the case of wet snuff)" [23]. Nicotine fulfils all the criteria of an addictive agent (including psychoactive effects, drug-reinforced behaviour, compulsive use, relapse after abstinence, physical dependence, and tolerance) by stimulating specialized receptors in the brain which produce both euphoric and sedative effects [24]. Individuals who have emotional dysfunctions or attention deficits are more likely to start smoking and less likely to quit. Nicotine has beneficial effects on attention, concentration, and mood in many smokers; these individuals may be depending on nicotine as a means of self-medication [25].

Are there important associated adverse health consequences of nicotine intake? The landmark work, Nicotine Safety and Toxicity, edited by Neal Benowitz, considered the potentially harmful effects of nicotine as well as its benefits [26]. After reviewing the evidence, the authors concluded that nicotine presents little if any cardiovascular risk, and that nicotine has not been shown to be carcinogenic. It is has been reported that nicotine may be potentially harmful during pregnancy, but probably less harmful than continued smoking [27-29]. There are data suggesting that nicotine may be beneficial in treating ulcerative colitis [30] and Tourette syndrome [31]. Other conditions for which nicotine is being considered as treatment include memory impairment, attention deficit disorder, depression, and Parkinson’s disease [32]. Regarding long-term use, even though nicotine is a potential toxin, it appears to be well-tolerated during weeks and months of nicotine medication therapy without evidence of serious adverse health effects [10]. Using the multi-criteria decision analysis method previously used by the Independent Scientific Committee on Drugs (ISCD) to rank the harms of drugs used in the UK, a working group of international nicotine experts convened by the ISCD considered the potential harms of a wide range of nicotine containing products based on sixteen parameters of harm to individuals and harm to others. Not only conventional cigarettes were judged to be by far the most harmful form of nicotine containing product, but e-cigarettes were ranked as similar in harm to nicotine patches [33]. By and large, nicotine *per se* does not cause much risk when separated from inhaling smoke.

### Current tobacco harm reduction products

Pharmaceutical nicotine products have been used as potential long-term cigarette substitutes. It has been reported that about 20 percent of smokers who quit with nicotine gum used it for more than one year, even though it was available only by prescription [34]. None of the currently available products deliver nicotine to the brain at a dose and rate similar to smoking. But this inadequacy is due to a philosophical aversion to nicotine addiction, not to technical inefficacy; a 1995 study found that high-dose transdermal nicotine was safe and effective for heavy smokers [35,36]. To be realistic alternatives, contemporary nicotine products need to be as readily available as cigarettes, competitively priced, socially acceptable and approved for regular long-term recreational use rather than as short-term cessation aids [22]. But these products would also be addictive.

**Figure 1:**
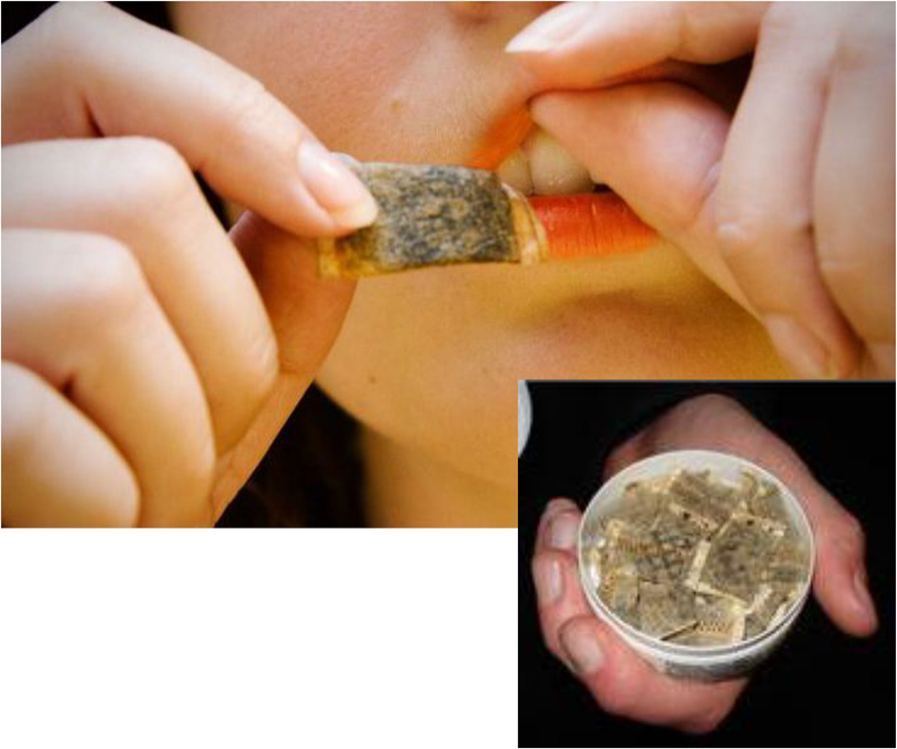
**Snus smoke**-**free tobacco.** Snus is an oral tobacco product that comes in a pouch of some sort, designed to be placed between the gums and upper lip. Snus is not chewed and requires no spitting. The standard pouch holds 1 gram of finely ground tobacco. Snus is regulated as a food in Sweden, and thus held to strict quality standards. Swedish snus was developed to greatly reduce TSNA content, and research shows that snus does not increase the risks of cancer of any type.

A convincing example of a successful THR strategy is that of Swedish snus. Snus is a type of finely ground moist snuff that delivers significant levels of nicotine (Figure 1). Snus does not produce any of the toxic combustion products and it is manufactured in a way that produces low levels of carcinogenic tobacco-specific nitrosamines (TSNAs) [37,38]. In Sweden, where snus has progressively replaced cigarette smoking over the past 20 years [35], substantial reductions in smoking prevalence have been reported [39]. Although Sweden’s tobacco control policies have undoubtedly contributed to this decline, the popularity of snus has played a major role. The much steeper decline in smoking prevalence observed among males than females is likely to be due to greater snus use in males [40]. Snus prevalence in Swedish males rose from 10% in 1976 to 23% in 2002 [41]. From the period 1990–1995 to the period 2002–2007, smoking prevalence decreased from 26 to 10% among men [42]Interestingly, the Swedish population prevalence of tobacco use has remained relatively steady at around 40%, but with 58% of daily tobacco users now taking snus instead of smoking cigarettes [43]. As a result of this, tobacco-related mortality in Sweden is among the lowest in the Western world [44]. Studies provide quantitative evidence that health risks of using snus is lower than smoking for lung, oral, and gastric cancers, for cardiovascular disease, and for all-cause mortality [45].

The Swedish experience has been replicated in Norway, which shares a border with Sweden and is culturally similar [46]. The 2005 California Tobacco Survey shows that smokers in that state are not receptive to using oral smokeless tobacco as a substitute for cigarette smoking [47]. One possible explanation for this phenomenon is that U.S. smoker’s perceptions of smokeless products are incorrect; indeed they are sceptical of the idea that snus is safer than cigarettes [48]. Misleading information disseminated by government agencies and non-profit health organizations has made American consumers [49,50] and health professionals [51] believe that smokeless tobacco is as harmful as, if not even more harmful than, smoking. Providing complete and truthful information could make U.S. smokers more receptive to switching to this much less harmful alternative.

The issue of abuse liability has been recently used by opponents of THR to warn about potential risks of smokeless tobacco products. Hatsukami et al. [52] concluded that smokeless tobacco appears to have slightly lower abuse liability, with possibly lower severity of addiction or dependence compared with smoking and greater ease of cessation. They also concluded that it may be possible that switching from cigarettes to smokeless tobacco would increase the potential for cessation from all tobacco products. Fagerström and Eissenberg came to similar conclusions in a recent comparison of dependence among smokers, smokeless tobacco users and users of medicinal nicotine [53]. Many former smokers in Sweden have quit through using snus, suggesting it may be a more effective cessation aid, and a more attractive long-term alternative to cigarettes, than pharmaceutical nicotine because its nicotine delivery and social aspects are similar to those of smoking [38,39,54]. Three small clinical trials support the role of smokeless tobacco as a cessation option for smokers. After reporting reduced TSNA exposure among smokers given an American snus product or Ariva (a dissolvable pellet), Mendoza-Baumgart et al. [55] concluded that this low-nitrosamine smokeless tobacco product has strong potential as a harm reduction tool. In 2010 Caldwell et al. [56] tested the acceptability of Swedish snus, nicotine gum and Zonnic (a pouch containing 4 milligrams of nicotine embedded in microcrystalline beads) among naive smokers in New Zealand. They reported that all three products significantly reduced craving for cigarettes, and all three enabled subjects to reduce their smoking significantly, with Zonnic and snus ranked higher than nicotine gum for both quitting and reducing smoking. Hence, it is not surprising that dissolvable tobacco products led to a significant reduction (approx. 40%) in cigarettes per day, no significant increases in total tobacco use, and significant increases in two measures of readiness to quit in a recent pilot randomized study [57].

The issue of abuse liability has been also used by anti THR supporters to warn about potential risks of e-cigarettes. However, in a recent randomized controlled trial of 300 smokers [58], only 26.9% of those who switched to e-cigs resulting in complete smoking abstinence were still using the product by the end of the observational period (week-52) with the 73.1% of users stopping vaping as well. That many regular vapers were able to free themselves also from the behavioral component of smoking that was being reproduced by vaping the product under investigation, indicates that the e-cigarettes are not very "addictive".

### Emerging tobacco harm reduction products: electronic cigarettes

Use of electronic cigarettes (E-cigs) may prove to be an even more attractive long-term alternative because of their similarities to smoking, including the hand-to-mouth repetitive motion and the visual cue of a smoke-like vapour.

**Figure 2:**
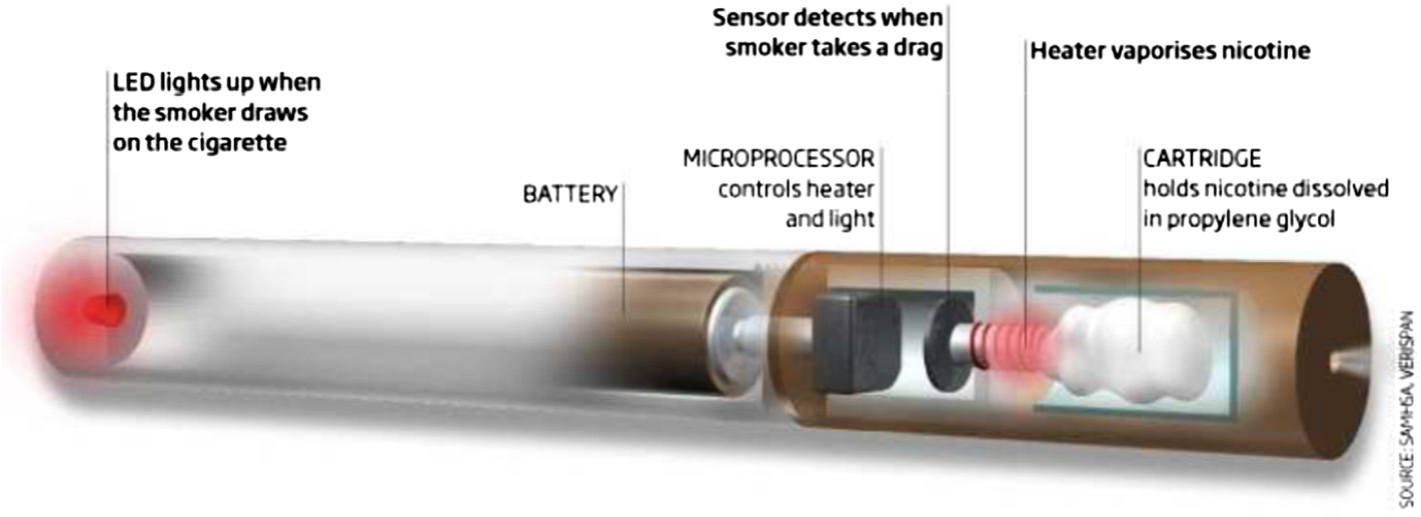
**Structure of a standard entry model electronic**-**cigarette (e**-**Cigarette).** The e-Cigarette is a battery-powered electronic nicotine delivery device (ENDD) resembling a cigarette designed for the purpose of providing inhaled doses of nicotine by way of a vaporized solution. The product provides a flavor and physical sensation similar to that of inhaled tobacco smoke, while no smoke or combustion is actually involved in its operation. It is composed of the following key components: (1) the inhaler – also known as 'cartridge’ (a disposable plastic mouthpiece - resembling a tobacco cigarette’s filter containing an absorbent material saturated with a liquid solution of propylene glycol and vegetable glycerin in which it may be dissolved nicotine); (2) the atomizing device (the heating element that vaporizes the liquid in the mouthpiece and generates the mist with each puff); (3) the battery component (the body of the device - resembling a tobacco cigarette – which houses a lithium-ion re-chargeable battery to power the atomizer). The body of the device also houses an electronic airflow sensor to automatically activate the heating element upon inhalation and to light up a red LED indicator to signal activation of the device with each puff. The LED indicator also signals low battery charge.

According to the WHO Study Group on Tobacco Product Regulation, E-cigs are categorized as electronic nicotine delivery systems (ENDS), devices designed for the purpose of nicotine delivery to the respiratory system where tobacco is not necessary for their operation [59]. Awareness and use of E-cigs has increased exponentially in the past four years. These devices, which are manufactured and sold by several different companies, consist of a lithium battery, electronic components, an atomizer, and a cartridge that holds a liquid solution composed of water, propylene glycol, flavourings, and nicotine (Figure 2). Their popularity appears to be related to the close similarities to smoking, the fact that they can be used in smoke-free places, the competitive price, and the perceived potential for harm reduction [60].

Cigarette smokers will keep smoking because of their addiction and when given the options of smoking or completely giving up nicotine, many will not give it up. This rigid dichotomous scheme may be now considered legacy of the past as many of them would be better off using nicotine in a low-risk form. E-cigs may be an additional tool for reducing tobacco related harm when used to target smokers for whom current cessation programmes have had only limited success [61]. E-cigs also may be attractive to inveterate smokers who consider their tobacco use a recreational habit that they wish to maintain in a more benign form, rather than a problem to be medically treated [62].

### Toxicological characterization of e-cigarettes

The available evidence indicates that e-cigarettes do not raise serious health concerns and can be considered a much safer alternative to conventional smoking [63-66].

Detailed toxicology characterization of e-cigarette liquid and vapour using gas chromatography mass spectrometry (GC-MS) demonstrates that their primary components are water, propylene glycol (PG), glycerin, and nicotine [67]. In an independent study, Laugesen tested E-cig mist for over 50 priority-listed cigarette smoke toxicants and found none [64]. This report only revealed traces (8.2 ng/g) of TSNAs in the “high” nicotine cartridge of a Ruyan brand E-cig. However, it must be noted that this amount is equal to the quantity reported to be present in a nicotine medicinal patch [61] (Table 1).

**Table 1:** **Summary data of maximum tabacco-specific nitrosamine levels in various cigarettes and nicotine-delivery products includine electronic cigarettes** (**ng**/**g**, **except for nicotine gum and patch that are ng**/**gum piece and ng**/**patch**) – ***Modified by Khan Z et al***. ***J Public Health Policy 2011***

**Product**	**NNN**	**NNK**	**NAT**	**NAB**
Nicorette gum (4 mg)	2.00	ND	ND	ND
NicoDerm CQ patch (4 mg)	ND	8.00	ND	ND
Electronic cigarettes	3.87	1.46	2.16	0.69
Swedish snus	980.00	180.00	790.00	60.00
Winston (full)	2200.00	580.00	560.00	25.00
Marlboro (full)	2900.00	960.00	2300.00	100.00
Camel (full)	2500.00	900.00	1700.00	91.00
Marlboro (ultra-light)	2900.00	750.00	1100.00	58.00

NNN, 4-(methylnitrosamino)-1-(3-pyridyl)-1-butanone; NNK, N’-nitrosonornicotine.

NAT, N’-nitrosoanatabine; NAB, N’-nitrosoanabasine.

ND, Not detected.

FDA-commissioned testing of e-cigarette cartridge fluids found diethylene glycol in one of the 18 e-cigarette cartridges tested [68]. Formaldehyde, acetaldehyde, and acroleine (potentially toxic carbonyl compounds) have been detected in e-cigarette vapour in 12 brands of e-cigarettes but at levels substantially lower than in cigarette smoke. These compounds may be formed by the oxidation of propylene glycol or glycerol when in contact with the heating coil.

Cahn and Siegel [61] reviewed the results of 16 laboratory analyses of E-cig liquid, including the FDA’s Report noted above . TSNAs were reported in two studies, but at trace levels, which are similar to those found in a nicotine patch, and, most importantly, about 500-fold to 1400-fold lower than TSNA levels measured in regular cigarettes (E-cigs containing only 0.07–0.2% of the TSNAs present in cigarettes) (Table 1).

It must be however noted that the e-cigarette industry is now adopting improved manufacturing standards. According to American e-liquid Manufacturing Standards Association (AESMA), liquids produced before 2013 were largely inaccurate, whereas newest products have substantially improved in term of purity, consistency and accuracy of nicotine content.

For example, [69] in a recent analysis of 20 refill liquids of 10 of the most popular brands have shown that the nicotine content in the bottles corresponded closely to the labels on the bottles with levels of nicotine degradation products being 1–2% for most samples. Also, this analysis did not detect ethylene glycol nor diethylene glycol; for several brands the levels of impurities were above the level set for nicotine products in the European Pharmacopoeia, but below the level likely to cause harm.

E-cigarette vapour contains a number of potentially toxic compounds. Testing on some devices has found tobacco-specific nitrosamines (TSNAs) [70]) and polycyclic aromatic hydrocarbons present in cartridge fluid, but generally in very low levels, similar to those in nicotine replacement therapy [64,68,71].

Cadmium, lead and nickel have also been detected in vapour but in trace levels only, comparable with levels found in Nicorette inhaler [72]. Metal and silicate particles were detected in fluid and vapour from e-cigarette cartomisers obtained from one manufacturer over several years, leading to exposure to amounts of these particles equal to or higher than users of tobacco cigarettes might typically experience [73].

In essence, these products appear to be much safer than tobacco cigarettes and comparable in toxicity to conventional nicotine replacement products. Of note, retailers have already sold hundreds of thousands of E-cigs with no evidence that these products have endangered anyone when used as directed. Although there is no indication that E-cigs are any more an immediate threat to public health and safety than traditional cigarettes, which are readily available to the public, the current data is insufficient to conclude that E-cigs are safe in absolute terms, and further studies are needed to comprehensively assess their safety, particularly in the long term.

### E-cigarette studies

The E-cig is a very hot topic that has generated considerable global debate, with authorities wanting to ban it or at least regulate it. Consequently, a formal demonstration supporting the efficacy and safety of these devices in clinical trials would be of utmost importance.

One of the earliest clinical trials of electronic cigarettes was conducted at the University of Auckland, New Zealand. Forty adult smokers of 10 or more cigarettes per day were randomized to use an E-cig containing 16 mg of nicotine or 0 mg of nicotine (placebo), a Nicorette nicotine inhalator, or their own brand cigarette. The 16 mg E-cig alleviated desire to smoke after overnight abstinence, was well tolerated and exhibited a pharmacokinetic profile more like the Nicorette inhalator than a tobacco cigarette [74]. In a small preliminary study of 16 smokers comparing two brands of E-cigs to the participants’ own brand, Eissenberg reported that 10 puffs from either brand delivered little to no nicotine compared with 10 puffs from the regular brand [75]. A response to this letter pointed out that each puff from an electronic cigarette delivers approximately 10% of the nicotine found in a puff of cigarette smoke [76]. Therefore E-cigs users need to take more puffs than smokers to raise blood nicotine levels. Final results of Eissenberg’s study for 32 participants confirmed that no measurable levels of nicotine or carbon monoxide were detected in E-cigs users. However, both brands effectively suppressed nicotine abstinence symptoms [77]. Recently Vansickel and Eissenberg studied blood nicotine levels and among subjects who used E-cigs according to a standard protocol after 12 hours of abstinence [78]. All subjects were former smokers who had quit smoking 11 months earlier and were veteran vapers. Blood nicotine levels increased from 2 nanograms per milliliter (ng/ml) at baseline to 10 ng/ml within 5 minutes of the first puff, and to 16 ng/ml at the end of the ad lib period of use. These levels are very similar to those produced by cigarette smoking, suggesting that a learning curve effect has to be taken into account when discussing clinical studies with E-cigs. Canadian researchers examined the reinforcing effects of E-cigs with and without nicotine on 11 volunteers. Participants reported a reduction in craving, regardless of the nicotine content [79]. Our recent smoking cessation study with a plastic nicotine-free inhalator, suggests that E-cigs can serve as an effective smoking replacement for some smokers, even if no nicotine is present [80].

Japanese researchers conducted a safety assessment of E-cigs with 32 smokers and found that following the treatment, no abnormal changes in blood pressure, hematological data, or blood chemistry and no severe adverse events were observed [62]. In a prospective proof-of-concept study, we monitored for 6 months possible modifications in smoking habits of 40 smokers not willing to quit who experimented with a 7.4 mg nicotine/cartridge E-cig [60]. Combined sustained smoking reduction and smoking abstinence was shown in 22/40 (55%) participants, with an overall 88% fall in cigs/day. Mouth and throat irritation, and dry cough were common, but diminished substantially by the end of the study. Participants’ perception and acceptance of the product was good.

That these results could be maintained for at least 24 months by adopting newer more efficient models as improved smoking sensation aids [81] indicates that these products have potential for efficient long-term substitution for smoking.

In a recent prospective 12-month randomized control design study (ECLAT study) we have just collected the data of E-cigs with 7.2 mg, 5.4 mg and 0 mg nicotine cartridges to measure smoking reduction or abstinence in 300 smokers unwilling to quit Declines in cig/day use and eCO levels were observed at each study visits in all three study groups (p,0.001 vs baseline), with no consistent differences among study groups. Smoking reduction was documented in 22.3% and 10.3% at week-12 and week-52 respectively. Complete abstinence from tobacco smoking was documented in 10.7% and 8.7% at week-12 and week-52 respectively. A substantial decrease in adverse events from baseline was observed and withdrawal symptoms were infrequently reported during the study [58].

In another recent randomized controlled trial, Bullen and coll. [82,83] randomised 657 adult smokers wanting to quit to 16 mg nicotine e-cigarettes (as needed), 21 mg nicotine patches (one per day), or placebo e-cigarettes (no nicotine, as needed) in a 4:4:1 ratio. Participants, who all lived in Auckland, New Zealand, could access the national Quitline (a telephone counselling service), but received no additional support. At 6 months, 7.3% participants in the nicotine e-cigarettes group had achieved biochemically verified abstinence, compared with 5.8% participants in the patches group, and 4.1% in the placebo e-cigarettes group. However, the statistical power was insufficient to conclude superiority of nicotine e-cigarettes to patches or to placebo e-cigarettes. As for other clinical studies with e-cigarettes, adverse events were very mild.

Several surveys [84-86] paint a picture of the typical e-cig consumer as a long-term smoker who tried repeatedly to quit. The median age of respondents ranges from late 30s to mid 40s. The percentage of respondents using e-cigs as a complete replacement for smoking ranged from 31% to 79%. Etter and Bullen found that 77% of daily users were former smokers, and 19% who were still daily smokers reduced their cigarettes per day from 25 to 15. The most-used flavour was tobacco, but 61% preferred various fruit flavours, coffee, vanilla, and chocolate [86]. Over 90% of respondents reported that their health has improved. When asked the main reason why they chose to use an e-cig, 64.6% selected “to continue to have a 'smoking’ experience, but with reduced health risks.” [85].

## Discussion

**Figure 3:**
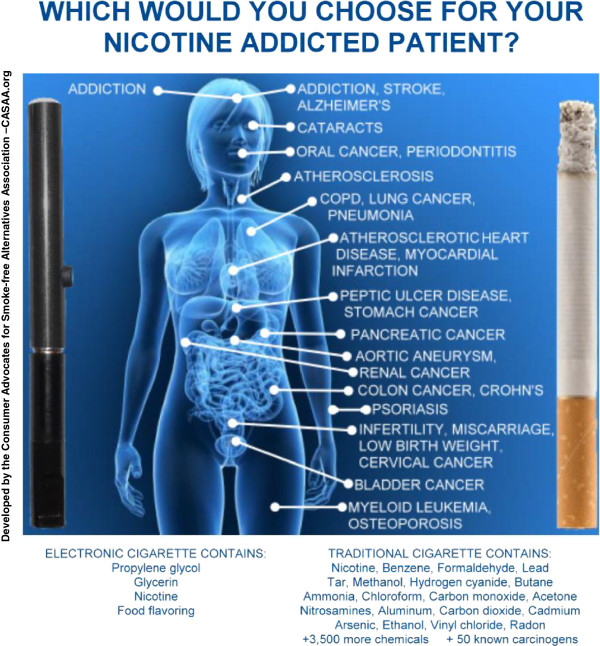
**Medical Infograph.** This Infograph compares the potential health risks of cigarette smoke with the health risks of vapor. Since e-cigarette liquid contains only propylene glycol, vegetable glycerin, flavorings, and nicotine, the resulting vapor is unlikely to present any more disease risk than medicinal nicotine products -- the risk of nicotine addiction. The many more toxic and carcinogenic ingredients in tobacco smoke are linked to numerous health problems.

E-cigs might be the most promising product for tobacco harm reduction to date. E-cigs deliver a nicotine vapour without the combustion products that are responsible for nearly all of smoking’s damaging effects (Figure 3). Temperatures of approximately 1.000 °C are generated with each puff of a lit cigarette, and thousands of toxic chemicals are produced during the combustion process [87]. In contrast, E-cigs use vaporization, rather than combustion, and the low operating temperature of the atomizer (up to 160 °C, depending on the model) does not emit cigarette toxicants [64]. Therefore, the health risks are likely to be similar to those from smokeless tobacco, which has approximately 1% of the mortality risk of smoking [49]. E-cigs may contain nicotine, which contributes to nicotine addiction and helps sustain tobacco use. However, if sufficient numbers of smokers can transfer their nicotine dependence to a less-harmful delivery method, millions of lives could be saved. The positive aspects of E-cigs appear to outweigh the negative aspects (Table 2).

**Table 2:** **Positive and negative aspects of e**-**cigarettes**

**Positive**	**Negative**
Beneficial effects on health (improved exercise tolerance, and less cough)	Small percent of the population is sensitive to propylene glycol (dry mouth and throat)
No tobacco smoke odor or bad breath	Some flavors (e.g. piña colada) have a lingering smell
Much less toxic than conventional cigarettes	Trace amounts of contaminants and metals present in some products
Mimics the “throat hit” sensation of inhaling smoke	“Throat hit” sensation dependent on hardware used and liquid composition
Replicates gestures or actions associated with smoking behavior	Equipment is heavier than traditional cigarette and puffing technique requires some training
Facilitates smoking abstinence	Not all users manage to quit smoking or reduce consumption of conventional cigarettes
Relieves withdrawal symptoms and craving for conventional cigarettes	Relief of withdrawal symptoms varies, affected by quality of equipment and nicotine strength of liquid
No risk to bystanders.	Due to few studies on potential risk to bystanders, some communities are outlawing indoor use
No ash, dirt, or burned clothes	Environmental concern about safe disposal of cartridges and batteries
Accessible prices (in the long run cheaper than conventional cigarettes)	The intricacies of their use and maintenance may hinder widespread adoption
Much improved self-regulatory framework by e-cigarettes industry	Impending medicinal regulation in many countries

Nonetheless, websites that provide information about the health risks of smokeless tobacco, have conflated these risks with the risks of smoking, misleading the public and smokers into believing that there is no potential for harm reduction by switching from smoked to smokeless products [49]. Yet, evidence continues to emerge that snus is an effective harm-reduction strategy [88]. Similar deceptive advice is being given to smokers who might be thinking about switching to E-cigs [89]. Foulds et al. [90] found that 78% of E-cig users they interviewed had not used any tobacco in the prior 30 days, but they still advised smokers to use proven treatments (e.g. counselling and FDA-approved drugs). This is a bizarre advice, in view of the fact that the subjects they interviewed had tried to quit smoking an average of nine times before taking up use of an E-cig, and two-thirds had tried to quit with an FDA-approved smoking cessation medication [90].

With the excuse of safeguarding public health and guiding regulatory strategies, extensive research on product design, toxicant exposure, abuse liability, youth initiation, and influence on cessation efforts has been advocated [91]. Thus it appears that the same tactics that are being used to keep less hazardous products such as snus from being widely adopted by smokers are being used to combat switching to E-cigs. None of the toxicological testing conducted in E-cigs has shown that users or bystanders are exposed to harmful levels of toxins or carcinogens. Any danger of toxicant contamination can be averted by forcing manufacturers to adopt a similar regulatory framework as for dietary supplements, provided that no claims are made about prevention or treatment of disease [92]. Under dietary supplement regulation, manufacturers must show that a product is not dangerous before introduction. Compliance with national good manufacturing practice policies would ensure that e-liquids are produced in a quality manner, do not contain contaminants or impurities, are accurately labelled, and are held under conditions to prevent adulteration. With regard to marketing and safety of e-cigarettes' electronics, batteries, and spare parts, these components are already regulated by existing directives.

There is no evidence that large numbers of non-smokers are purchasing or will purchase E-cigs and become addicted to nicotine. E-cigs eliminate exposure to the smoke toxicants responsible for nearly all smoking-related diseases. Thus even if 50% of the non-smoking population should decide to addict itself to nicotine via an E-cig, the associated disease risks, if any, would be minimal. Thus, “abuse liability” is a moot point in this context.

Furthermore, E-cigs represent a middle ground between nicotine maintenance using the most deadly of delivery mechanism, smoking, and the nicotine abstinence demanded by the tobacco control community [93]. Fears that smokers who “might have quit altogether” will instead switch to snus or E-cigs is further evidence that the tobacco control community believes that total abstinence is something that all smokers will eventually embrace, and perhaps come to love. However, research shows that many smokers are dependent on the beneficial effects of nicotine to combat symptoms of underlying conditions [10] and that long-term nicotine abstinence may result in long-term discomfort for many smokers [94].

### Summary

The dream of a tobacco-free, nicotine-free world is just that—a dream. Nicotine’s beneficial effects include correcting problems with concentration, attention and memory, as well as improving symptoms of mood impairments. Keeping such disabilities at bay right now can be much stronger motivation to continue using nicotine than any threats of diseases that may strike years and years in the future.

Nicotine’s beneficial effects can be controlled, and the detrimental effects of the smoky delivery system can be attenuated, by providing the drug via less hazardous delivery systems. Although more research is needed, e-cigs appear to be effective cigarette substitutes for inveterate smokers, and the health improvements enjoyed by switchers do not differ from those enjoyed by tobacco/nicotine abstainers.

It is of paramount importance that government and trusted health authorities provide accurate and truthful information about the relative risks of smoking and alternatives to smoking. If the public continues to be misled about the risks of THR products, millions of smokers will be dissuaded from switching to these much less hazardous alternatives. One of us recently wrote that, “It’s time to be honest with the 50 million Americans, and hundreds of millions around the world, who use tobacco. The benefits they get from tobacco are very real. It’s time to abandon the myth that tobacco is devoid of benefits, and to focus on how we can help smokers continue to derive those benefits with a safer delivery system” [95].

In the absence of regulatory standards, it is important that currently marketed products are of high quality. For example, the hardware should be reliable and should produce vapour consistently. The liquids should be manufactured under sanitary conditions and use pharmaceutical grade ingredients, and labels should contain a list of all ingredients and an accurate and standardized description of the nicotine content.

According to a recent article by CDC researchers, the proportion of U.S. adults who have ever used electronic cigarettes more than quadrupled from 0.6% in 2009 to 2.7% in 2010 with an estimated number of current electronic cigarette users of about 2.5 million [96]. Although rigorous studies are required to establish THR potential and long term safety of electronic cigarettes, these figures clearly suggest that smokers are finding these products helpful. If they were ineffective one would not expect the market to take off as it is. Most importantly, even if this THR product proves to be effective for only 25% of the smoking population, it could save millions of lives world-wide over the next ten years.

## Competing interests

R.P. is Professor of Medicine and he is supported by the University of Catania, Italy. He has received lecture fees and research funding from GlaxoSmithKline and Pfizer, manufacturers of stop smoking mediactions. He has also served as a consultant for Pfizer and Arbi Group Srl (Milano, Italy), the distributor of Categoria™ e-Cigarettes. R.P.’s research on electronic cigarettes is currently supported by LIAF (Lega Italiana AntiFumo). B.R.’s research is supported by unrestricted grants from tobacco manufacturers to the University of Louisville, and by the Kentucky Research Challenge Trust Fund. P.C. and C.R. are Assistant Professors and they are supported by the University of Catania, Italy. M.M is researcher and she is supported by the University of Catania, Italy. They have no relevant conflict of interest to declare in relation to this work.

## Authors’ contributions

All authors revised the article critically for important intellectual content and approved its final version.
